# Maternal Age at Delivery Is Associated with an Epigenetic Signature in Both Newborns and Adults

**DOI:** 10.1371/journal.pone.0156361

**Published:** 2016-07-06

**Authors:** Christina A. Markunas, Allen J. Wilcox, Zongli Xu, Bonnie R. Joubert, Sophia Harlid, Vijayalakshmi Panduri, Siri E. Håberg, Wenche Nystad, Stephanie J. London, Dale P. Sandler, Rolv T. Lie, Paul A. Wade, Jack A. Taylor

**Affiliations:** 1 Epidemiology Branch, National Institute of Environmental Health Sciences, NIH, Research Triangle Park, North Carolina, United States of America; 2 Epigenetics and Stem Cell Biology Laboratory, National Institute of Environmental Health Sciences, NIH, Research Triangle Park, North Carolina, United States of America; 3 Norwegian Institute of Public Health, Oslo, Norway; 4 Department of Global Public Health and Primary Care, University of Bergen, Bergen, Norway; University of Southampton, UNITED KINGDOM

## Abstract

Offspring of older mothers are at increased risk of adverse birth outcomes, childhood cancers, type 1 diabetes, and neurodevelopmental disorders. The underlying biologic mechanisms for most of these associations remain obscure. One possibility is that maternal aging may produce lasting changes in the epigenetic features of a child’s DNA. To test this, we explored the association of mothers’ age at pregnancy with methylation in her offspring, using blood samples from 890 Norwegian newborns and measuring DNA methylation at more than 450,000 CpG sites across the genome. We examined replication of a maternal-age finding in an independent group of 1062 Norwegian newborns, and then in 200 US middle-aged women. Older maternal age was significantly associated with reduced methylation at four adjacent CpGs near the 2^nd^ exon of *KLHL35* in newborns (p-values ranging from 3x10^-6^ to 8x10^-7^). These associations were replicated in the independent set of newborns, and replicated again in women 40 to 60 years after their birth. This study provides the first example of parental age permanently affecting the epigenetic profile of offspring. While the specific functions of the affected gene are unknown, this finding opens the possibility that a mother’s age at pregnancy could affect her child’s health through epigenetic mechanisms.

## Introduction

Advanced maternal age during pregnancy has been associated with adverse birth outcomes [1–4] as well as health problems in children (childhood cancer [5], type 1 diabetes [6], and neurodevelopmental disorders [7,8]). The biologic mechanisms underlying most of these associations remain unknown. One mechanism by which maternal age could influence the health of offspring is through epigenetic modifications such as DNA methylation. DNA methylation refers to the addition of a methyl group to the 5’ position of a cytosine at cytosine-guanine dinucleotides (CpGs) and has been associated with a growing number of diseases.

During development, the parental genomes are completely demethylated between fertilization and implantation. New methylation marks start to be established around the time of blastocyst implantation, and continue throughout fetal growth [9]. Some of the only exceptions are a relatively small number of imprinted loci. Placement of the early epigenetic marks is of growing interest because of their potential for modulating gene transcription, development, and disease risk. In particular, DNA methylation has been proposed [10] as a mechanism by which parental or fetal exposure could determine subsequent risk of adult diseases (the “developmental origins of disease” hypothesis [11]). The mechanisms by which remethylation is controlled during development at various sites across the genome are still unknown; there are very few documented examples of parental exposures or direct fetal exposure *in utero* that lead to persistent epigenetic change in the offspring [12,13].

It is well established that as people age, there are changes in the levels of DNA methylation in blood and various other tissues (for example [14]). However, the epigenetic consequences of advanced maternal age in offspring have been little explored. Support for an epigenetic effect includes reports of age-related gene-expression changes in both mouse [15] and human oocytes [16]; some of these differentially expressed genes play a role in chromatin structure and DNA methylation. Age-related changes in DNA methylation have also been observed in mouse oocytes and mouse and human sperm [17–19]. To date, only one study has investigated the relationship between parental age and DNA methylation in offspring [20]. Although a number of maternal age-related DNA methylation changes were reported, the study was limited by a small sample size, the use of a low-density DNA methylation microarray, and the lack of an independent population for replication of their findings. Thus, the possible effects of maternal age on the newborn epigenome, and the persistence of such effects if they exist, remain mostly unexplored.

## Results

**Fig 1:**
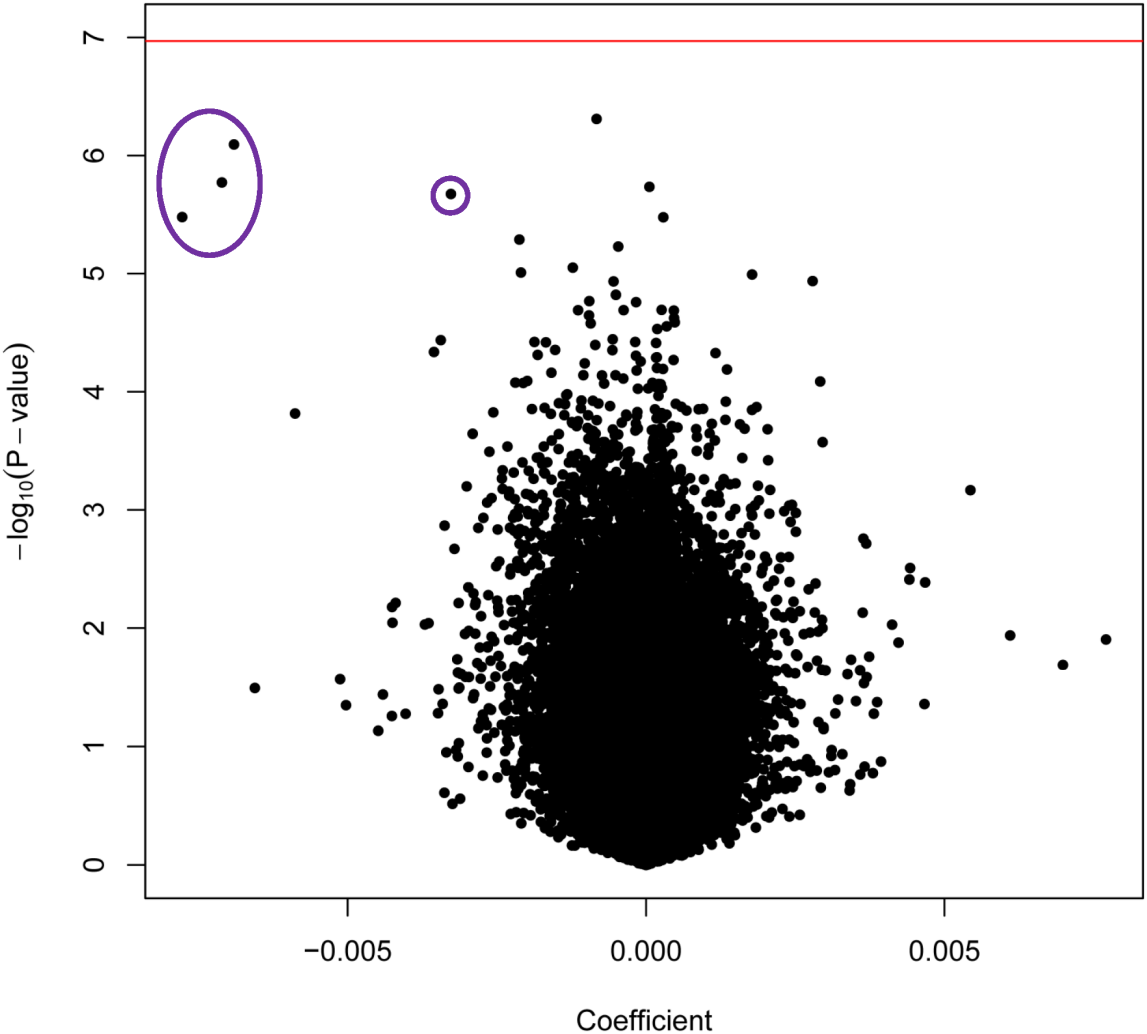
Epigenome-wide association results of maternal age in NFCS newborns. Volcano plot showing the relationship between the effect magnitude (coefficient) and strength of association (p-value) for Model1. The red horizontal line denotes the strict threshold for epigenome-wide significance based on a conservative Bonferroni correction for 465525 tests (p < 1.07x10^-7^). The four CpGs that are circled in purple are near the second exon of the gene, *KLHL35*.

**Fig 2:**
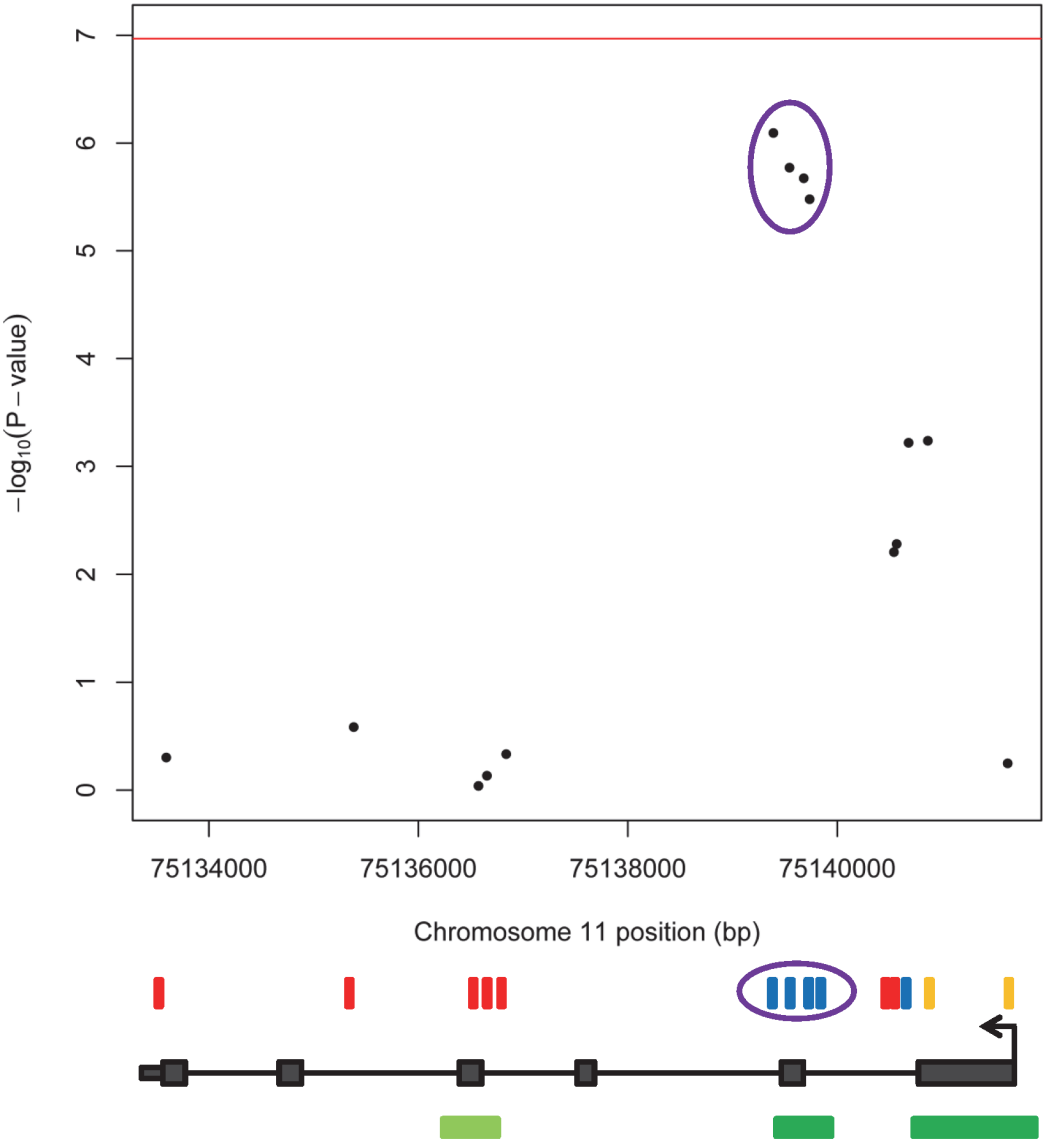
*KLHL35* methylation association results in NFCS. All *KLHL35* CpGs present on the Illumina 450K chip are shown as rectangles above the gene diagram. Each CpG is colored according to its mean absolute β-value adjusted for technical factors: red (70–100%), blue (30–70%), and orange (0–30%). The four CpGs that are circled in purple are near the second exon and show the strongest associations. The green rectangles below the gene diagram mark the CpG islands (UCSC, GC content ≥ 50%, length > 200 bp with islands < 300 bp shown in light green, Observed/Expected CpGs > 0.6). The red horizontal line denotes the strict threshold for epigenome-wide significance based on a conservative Bonferroni correction for 465525 tests (p < 1.07x10^-7^).

**Fig 3:**
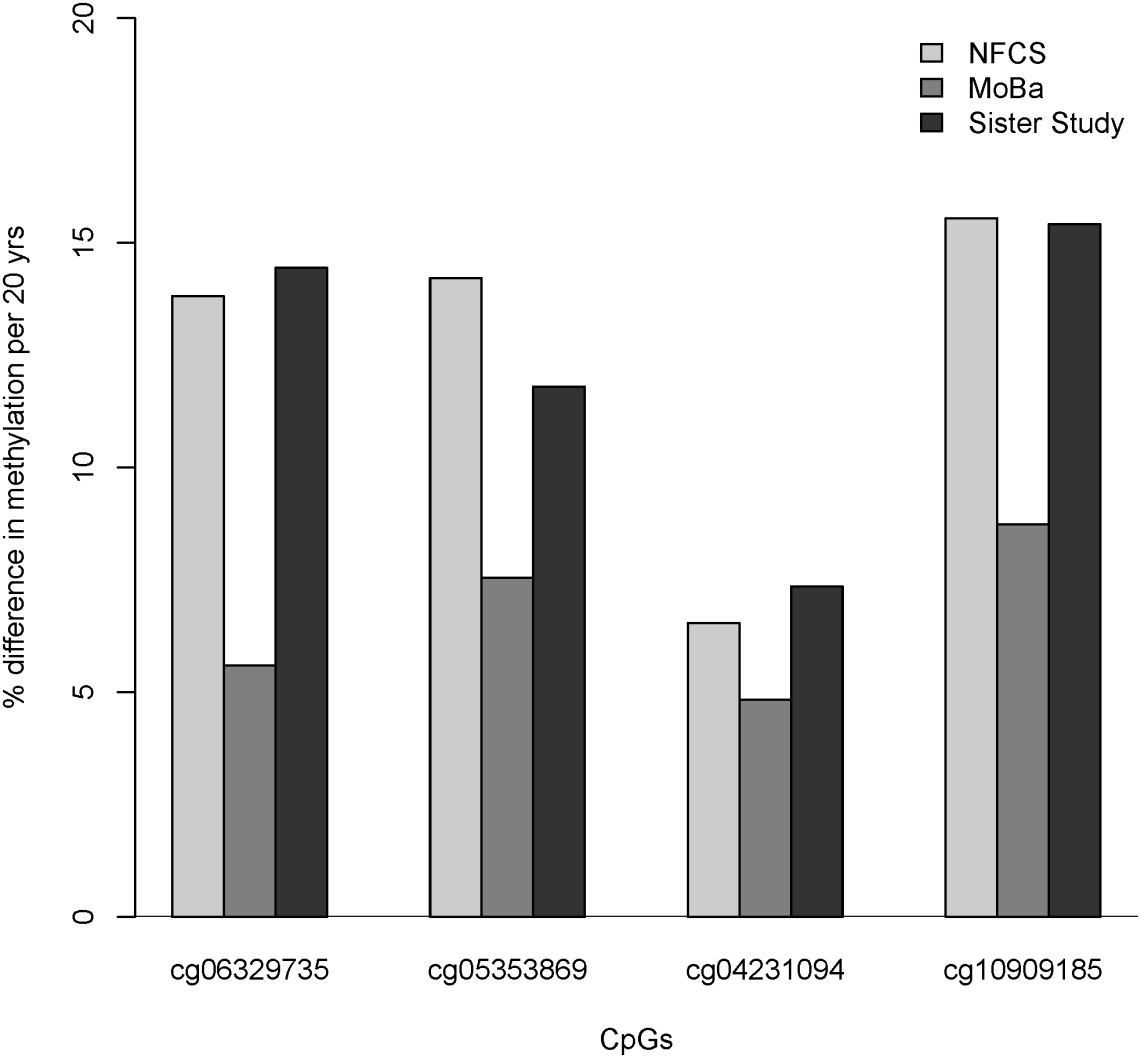
Maternal age-related DNA methylation changes in newborns (NFCS and MoBa) and adults (Sister Study) per 20 years. Methylation at each CpG (β-value) was tested for association with maternal age and the β coefficients from the regression analysis were converted into the percent difference in methylation per 20 years (maternal age). The covariates included in Model1 differed slightly across the NFCS, MoBa, and the Sister Study analyses (S1 Text).

In this epigenome-wide association study (EWAS), maternal age at delivery was associated with decreased methylation at four sites in the newborn epigenome. Using samples from a study of Norwegian newborns (NFCS), we identified four adjacent CpGs near exon 2 of *KLHL35* (kelch-like family member 35) with P-values ranging from 3.3x10^-6^ to 8.1x10^-7^ (Figs 1 and 2; S2 Table: Model1; S2–S4 Figs). Although not significant after strict Bonferroni correction for 465,525 tests (genome-wide threshold p < 1.07x10^-7^; S5 Fig), these CpGs showed surprisingly large effect sizes (Fig 1; S2 and S3 Tables): The mean methylation level (average β-values) for these four CpGs was 40%. A 20-year increase in maternal age would be estimated to decrease average absolute methylation at these sites by roughly 12.5% (Fig 3), representing a 35% relative decrease in methylation. We found no corresponding effect of paternal age on methylation levels at these sites (S4 Table).

The Adkins study of maternal age and methylation (based on 168 newborns) does not allow us to attempt to replicate our findings, as the four CpGs identified in our study were not covered by the Illumina 27K array used by Adkins [20]. None of the associations reported by Adkins were replicated in our data.

We sought replication of the four KLHL35 CpGs associated with maternal age in an independent study of Norwegian newborns (MoBa). Although effect sizes were somewhat smaller, all four CpGs near exon 2 showed statistically significant decreases in methylation (p<0.05, with 3 of the 4 meeting an appropriate Bonferroni correction; Fig 3 and S5 Table).

To assess the long-term persistence of these maternal-age effects, we conducted a second replication of the candidate *KLHL35* CpGs in a smaller sample of adult women from the Sister Study [21]. All four CpGs near exon 2 showed reduced methylation with advanced maternal age (p<0.05, although none met an appropriate Bonferroni correction). Moreover, the reduction in methylation levels were remarkably similar to those found in the original analysis of NFCS newborns (Fig 3 and S5 Table: Model1).

## Discussion

We have identified a distinctive pattern of reduced DNA methylation in the blood of newborn infants that is linked to the mother’s age at delivery. Importantly, this pattern at four CpG sites in the *KLHL35* gene was replicated in an independent sample of Norwegian newborns, and again in a sample of adult US women. Although we were unable to obtain serial biologic samples from the same individuals, the identification of the same maternal age-related DNA methylation changes in both newborns and adults suggests that these changes likely persist from birth for more than 40 years. To date, there are very few examples of parental exposures before conception, or even direct fetal exposure in utero, leading to a persistent epigenetic change throughout life [12,13]. While the reported maternal age association could be due to unmeasured or uncontrolled confounding, we have adjusted for several factors in our statistical model, including infant’s birth weight, maternal alcohol use, maternal smoking, maternal education, and parity. Additional factors such as gestational age, maternal body mass index, multivitamin use, dietary folate, and folic acid supplement use during pregnancy were explored but not included in the model as potential confounders as they were not associated with maternal age at delivery (p<0.05).

A mechanism by which maternal age might lead to persistent epigenetic differences in the offspring is unclear. These changes could be due to epigenetic inheritance of an altered maternal chromatin state. Perhaps more likely is that an altered *in utero* environment affects the re-establishment of DNA methylation marks within *KLHL35* after the initial wave of demethylation. In the absence of experimental data, it is unclear whether the altered DNA methylation state is biologically significant, influencing gene expression or chromatin structure. While CpGs at only one gene were found to show this methylation pattern with maternal age, there are vast numbers of CpG sites not captured by the 450K panel. If these sites are affected by maternal age, it seems plausible that other sites could also be affected. Further, our study only examined one form of epigenetic mechanism, thus there remains the possibility that other forms of epigenetic modifications, such as histone modifications or miRNAs, could also be influenced by maternal age.

Not much is known about *KLHL35*. This gene is part of the kelch-like gene family [22] and has been associated with cancer. In particular, *KLHL35* is hypermethylated in hepatocellular carcinoma [23], renal cell carcinoma [24], and various other cancers, based on data from The Cancer Genome Atlas [14]. In addition, RNAi knockdown of *KLHL35* in human embryonic kidney cells results in an anchorage-independent growth advantage [24]. Taken together, epigenetic dysregulation of *KLHL35* appears to be a feature shared across multiple cancers.

An unusual feature of the implicated CpG sites in the *KLHL35* gene is that they exhibit intermediate levels of absolute DNA methylation (Adjusted β-values in NFCS range from: 0.30 to 0.54). CpG methylation levels are more typically near 100% or zero percent at given sites. One way a CpG site might have intermediate levels of methylation is if one parental allele is imprinted (with the different methylation levels in the maternal and paternal alleles averaging out to an apparent intermediate level of methylation at the site). However, the *KLHL35* gene does not seem to be one of the imprinted genes. Known examples of maternally imprinted loci escape the wave of demethylation that occurs following fertilization [9]. In contrast, the exon 2 region of *KLHL35* is likely susceptible to epigenetic reprogramming after fertilization, since no DNA methylation is observed in single blastocysts for the single CpG site in this region (cg05353869) with data in GSE51239 (see S1 Text, for details) [25]. If there is differential methylation of parental alleles in this region, traditional imprinting is unlikely to explain the observed relationship.

In sum, we find a novel and robust pattern of gene methylation associated with mother’s age. This epigenetic change is apparently persistent (detectable > 40 years after birth), and one of only a few examples of epigenetic modifications associated with the prenatal environment. The relationship between epigenetic changes in the newborn and subsequent health outcomes remains to be seen.

## Material and Methods

### Discovery population

The discovery population was made up of newborns from the Norway Facial Clefts Study (NFCS), which has been previously described in detail [26]. Briefly, NFCS is a national population-based case-control study of facial clefts (incomplete fusion of the lip and/or palate during development). In the current study, a subset of 418 facial cleft cases and 480 controls were selected based on DNA availability. Details regarding sample collection and study population are provided (S1 Text, S1 Table, S1 Fig). The study, including the consent procedure, was approved by the Norwegian Data Inspectorate and Regional Medical Ethics Committee of Western Norway, and written informed consent was obtained from both the mother and father for the child.

### Replication populations: newborns and adults

Replication of selected findings was conducted using data from an independent pregnancy cohort, the Norwegian Mother and Child Cohort Study (MoBa) conducted by the Norwegian Institute of Public Health [27,28]. Babies in the methylation analysis were selected from a sub-study originally designed to examine the relationship between maternal plasma folate during pregnancy and childhood asthma at three years [29]. Illumina Human Methylation450K data for selected CpG sites from 1062 umbilical cord blood samples [30] were used for the replication analysis. Information regarding maternal age and other characteristics of mothers and infants in the replication study are provided (S1 Text, S1 Fig). The MoBa study, including consent procedure, was approved by the Regional Committee for Ethics in Medical Research, the Norwegian Data Inspectorate and the Institutional Review Board of the National Institute of Environmental Health Sciences (NIEHS), USA, and written informed consent was provided by all mothers participating.

In order to explore whether selected maternal-age-related changes in DNA methylation persist into adulthood, we used data from the Sister Study, a nationwide prospective US cohort of women having a sister with breast cancer. Illumina Human Methylation450K data from 200 adult women [21] originally selected based on *in utero* exposure to diethystillbestrol (DES) were used in the analysis. Additional details regarding maternal and participant characteristics are provided (S1 Text, S1 Fig). Informed written consent was obtained from all participants prior to participation. The study, including consent procedure, was approved by the Institutional Review Boards of the National Institute of Environmental Health Sciences (NIEHS), National Institutes of Health, and the Copernicus Group (http://www.cgirb.com/irb-services/).

### Analysis: DNA methylation data

Detailed descriptions of DNA methylation data generation (Illumina HumanMethylation450 beadchips), quality assessment, and pre-processing for all three data sets have been previously published (NFCS [31], with slight modifications in data processing described in S1 Text; the Sister Study [21], and MoBa [32]). We corrected for batch in MoBa with ComBat [33], using the SVA package in R.

### Initial analysis: Epigenome-wide association in NFCS

Robust linear regression was used to test the association between maternal age at delivery and the methylation level (β-value) at each CpG site (R package, MASS [34]). The primary model adjusted for technical factors (96-well plate, bisulfite conversion efficiency, infant’s birth year), facial cleft status (control, cleft lip with or without cleft palate, and cleft palate only), infant sex, and several potential confounders (infant birth weight, maternal alcohol use, maternal smoking, maternal education, and parity). Details regarding the evaluation of additional models and covariate selection is provided (S1 Text). A highly conservative Bonferroni correction for 465,525 tests was used as the criterion for epigenome-wide significance (p < 1.07 x 10^−7^). All analyses were conducted using R version 3.0.1.

### Replication analyses: newborns and adults

Findings from the discovery population were replicated in independent samples of infants (from the MoBa study) and adult women (from the Sister Study) using robust linear regression to test the association between maternal age at delivery and methylation levels (β-values) at the selected CpG sites (R package, MASS [34]). Additional information regarding analytic models is provided in S1 Text. A conservative Bonferroni correction was used to adjust for multiple testing.

## Supporting Information

S1 Fig Maternal age at delivery distribution in discovery and replication populations. A) NFCS newborns (N = 890), B) Sister Study 450K adults (N = 200), and C) MoBa newborns (N = 1062).
(TIFF)

S2 Fig Epigenome-wide association results for Model1 in NFCS newborns. Manhattan plot where the red horizontal line denotes the strict threshold for epigenome-wide significance based on a conservative Bonferroni correction for 465525 tests (p < 1.07x10^-7^). The four CpGs that are circled in purple are near the second exon of the gene, *KLHL35*.
(TIFF)

S3 Fig β-value distributions of select *KLHL35* CpGs in NFCS newborns. A) cg06329735, B) cg05353869, C) cg04231094, and D) cg10909185. β-values are adjusted for technical factors: batch, bisulfite conversion efficiency, and infant’s birth year.
(TIFF)

S4 Fig Relationship between maternal age at delivery and DNA methylation β-values for CpGs of interest. A) cg04231094, B) cg05353869, C) cg06329735, and D) cg10909185. β-values are adjusted for technical factors: batch, bisulfite conversion efficiency, and infant’s birth year.
(TIFF)

S5 Fig Quantile-Quantile (Q-Q) plot for epigenome-wide association study in NFCS. Plots the observed (Model1) versus expected -log_10_(p-values) under the null hypothesis of no association.
(TIFF)

S6 Fig Comparison of Model0 and Model1 maternal age results in NFCS newborns. Model0: Methylation (β-value) = maternal age + batch + bisulfite conversion efficiency + infant’s birth year; Model1: Methylation (β-value) = maternal age + cleft + infant’s sex + batch + bisulfite conversion efficiency + infant’s birth year + infant’s birth weight + maternal alcohol use + maternal smoking + maternal education + parity, A) Comparison of the maternal age coefficient in Model0 versus Model1, B) Comparison of the maternal age –log10(P-value) in Model0 versus Model1.
(TIFF)

S7 Fig Comparison of Model1 and Model2 maternal age results in NFCS newborns. Model1: Methylation (β-value) = maternal age + cleft + infant’s sex + batch + bisulfite conversion efficiency + infant’s birth year + infant’s birth weight + maternal alcohol use + maternal smoking + maternal education + parity; Model2: Methylation (β-value) = maternal age + cleft + infant’s sex + batch + bisulfite conversion efficiency + infant’s birth year + infant’s birth weight + maternal alcohol use + maternal smoking + maternal education + parity + six leukocyte proportions (CD8+ T cells, CD4+ T cells, Natural killer cells, B cells, Monocytes, Granulocytes), A) Comparison of the maternal age coefficient in Model1 versus Model2, B) Comparison of the maternal age –log10(P-value) in Model1 versus Model2.
(TIFF)

S1 Table Characteristics of mothers and infants in NFCS^a^. (DOCX)

S2 Table Detailed *KLHL35* results across models^a^ in NFCS. (DOCX)

S3 Table Detailed *KLHL35* Model1 results in NFCS—maternal age at delivery as quartiles. (DOCX)

S4 Table Select *KLHL35* results for Model1 plus paternal age using the Norway Facial Clefts Study. (DOCX)

S5 Table Replication of *KLHL35* maternal-age related DNA methylation changes in newborns and adults: a comparison of models^a^. (DOCX)

S1 Text Supplemental Methods and References. (DOCX)
