# Supplementary figures and images for: Maternal Age at Delivery Is Associated with an Epigenetic Signature in Both Newborns and Adults
Source: PLoS One. 2016 Jul 6;11(7):e0156361. doi: 10.1371/journal.pone.0156361 (PMC4934688; doi:10.1371/journal.pone.0156361)

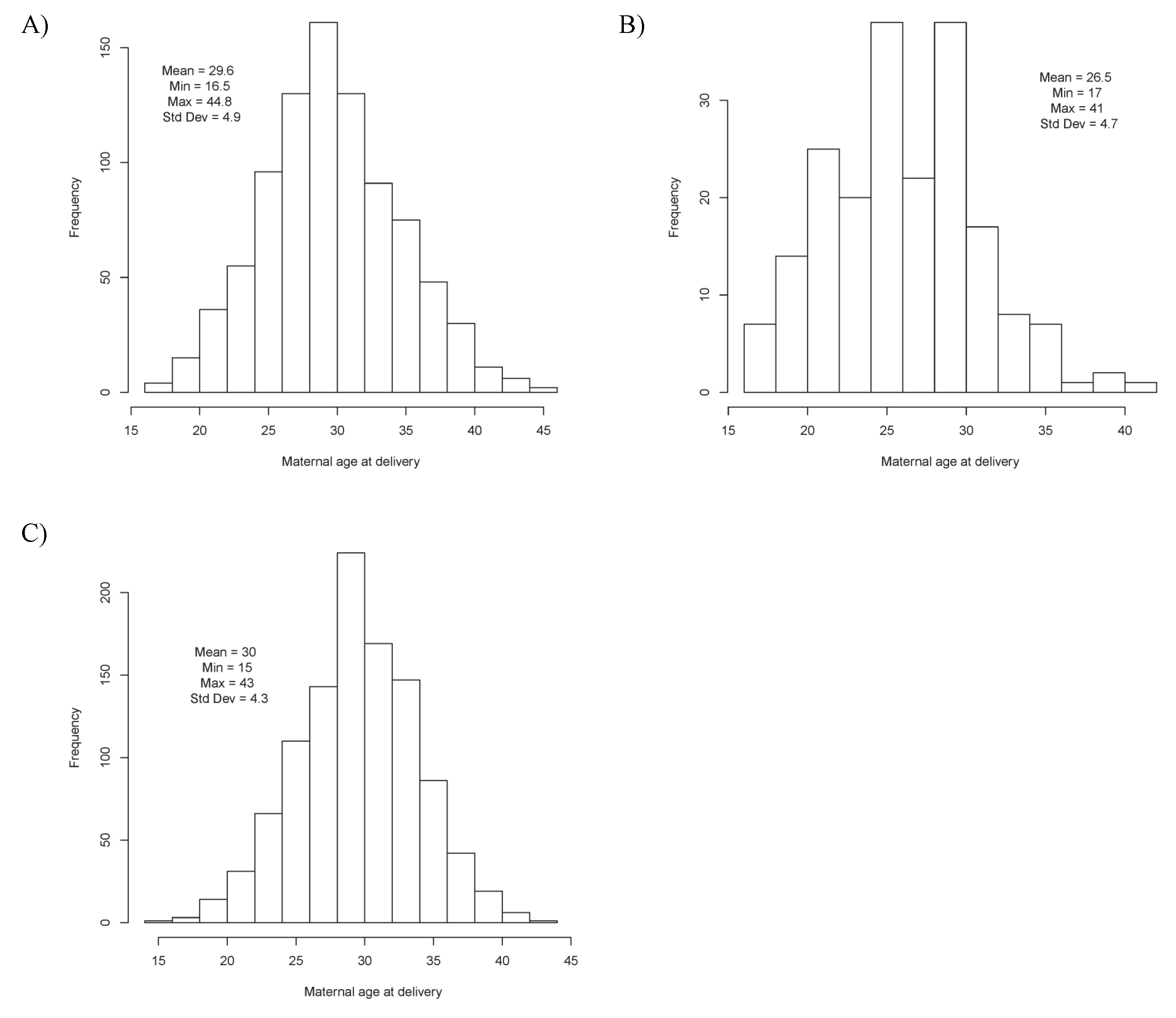

Supplement: S1 Fig — A) NFCS newborns (N = 890), B) Sister Study 450K adults (N = 200), and C) MoBa newborns (N = 1062). (TIFF) [file pone.0156361.s001.tiff]

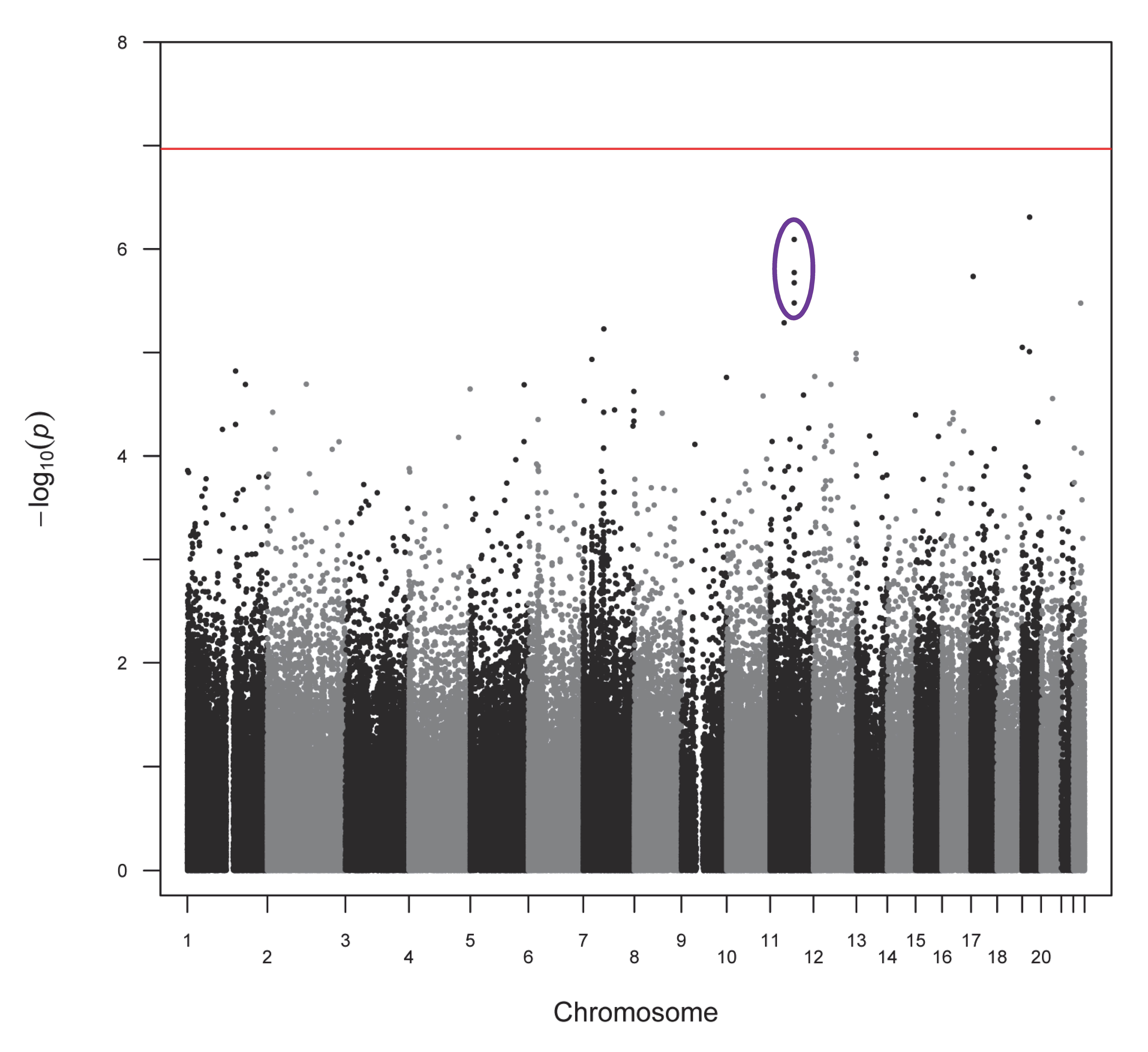

Supplement: S2 Fig — Manhattan plot where the red horizontal line denotes the strict threshold for epigenome-wide significance based on a conservative Bonferroni correction for 465525 tests (p < 1.07x10^-7^). The four CpGs that are circled in purple are near the second exon of the gene, KLHL35. (TIFF) [file pone.0156361.s002.tiff]

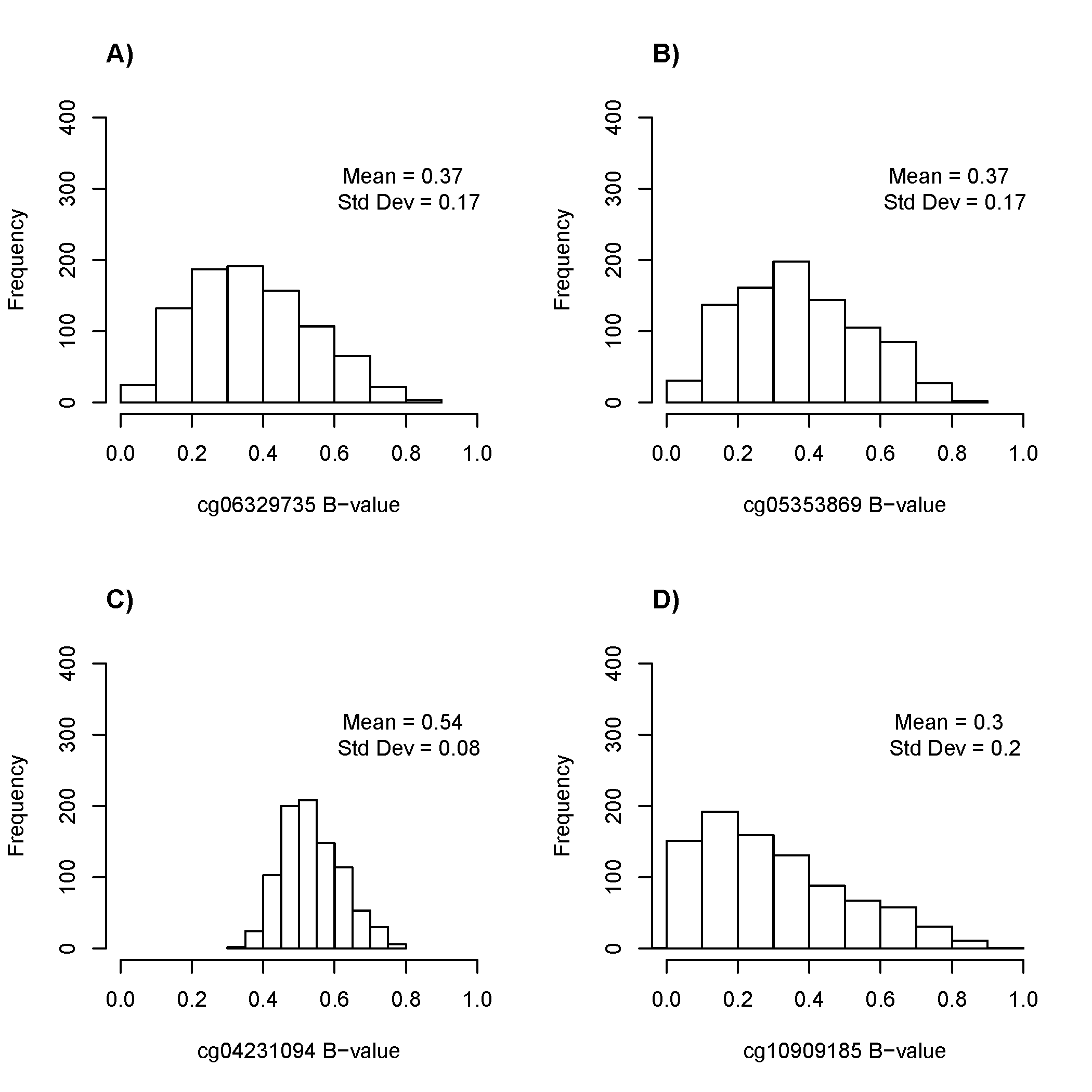

Supplement: S3 Fig — A) cg06329735, B) cg05353869, C) cg04231094, and D) cg10909185. β-values are adjusted for technical factors: batch, bisulfite conversion efficiency, and infant’s birth year. (TIFF) [file pone.0156361.s003.tiff]

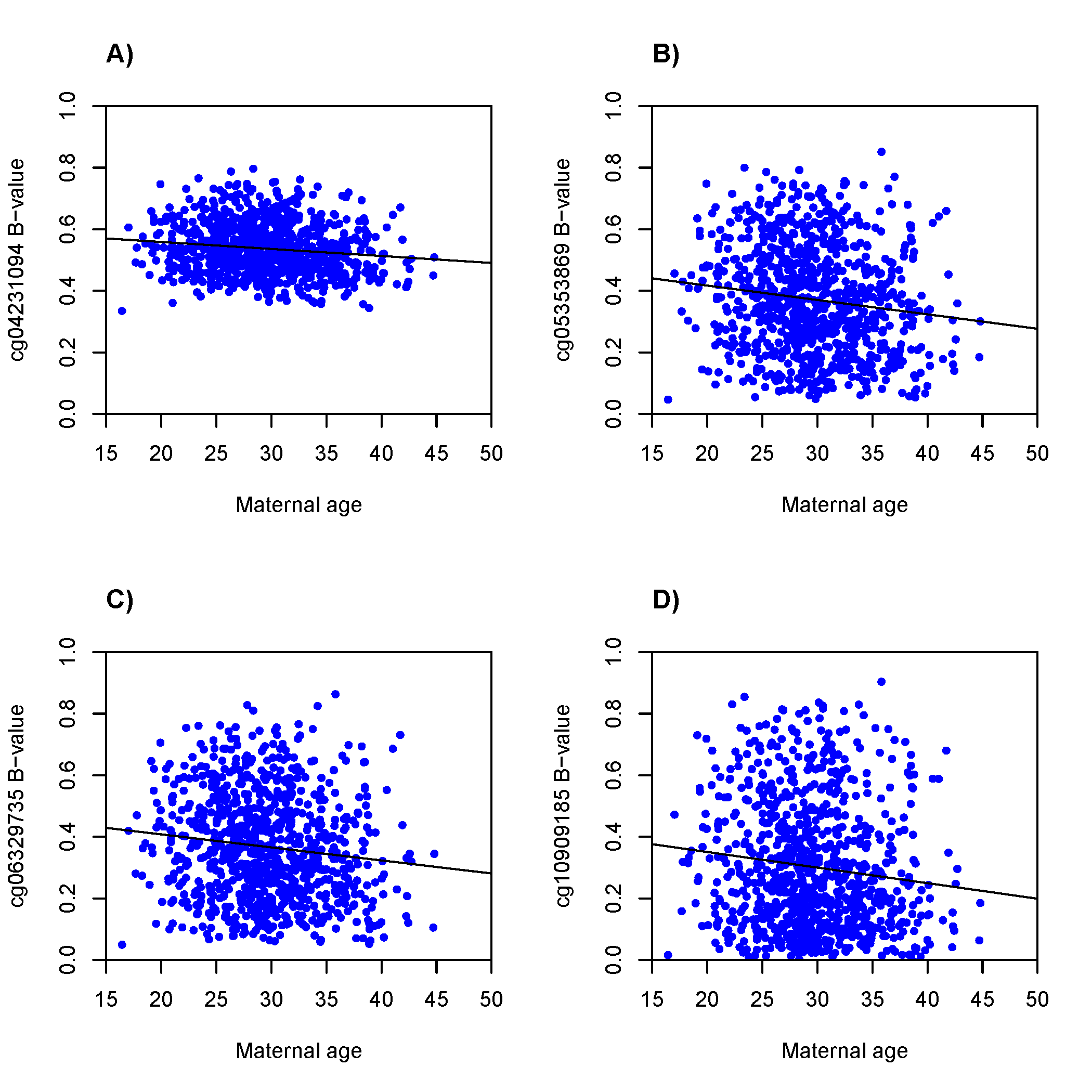

Supplement: S4 Fig — A) cg04231094, B) cg05353869, C) cg06329735, and D) cg10909185. β-values are adjusted for technical factors: batch, bisulfite conversion efficiency, and infant’s birth year. (TIFF) [file pone.0156361.s004.tiff]

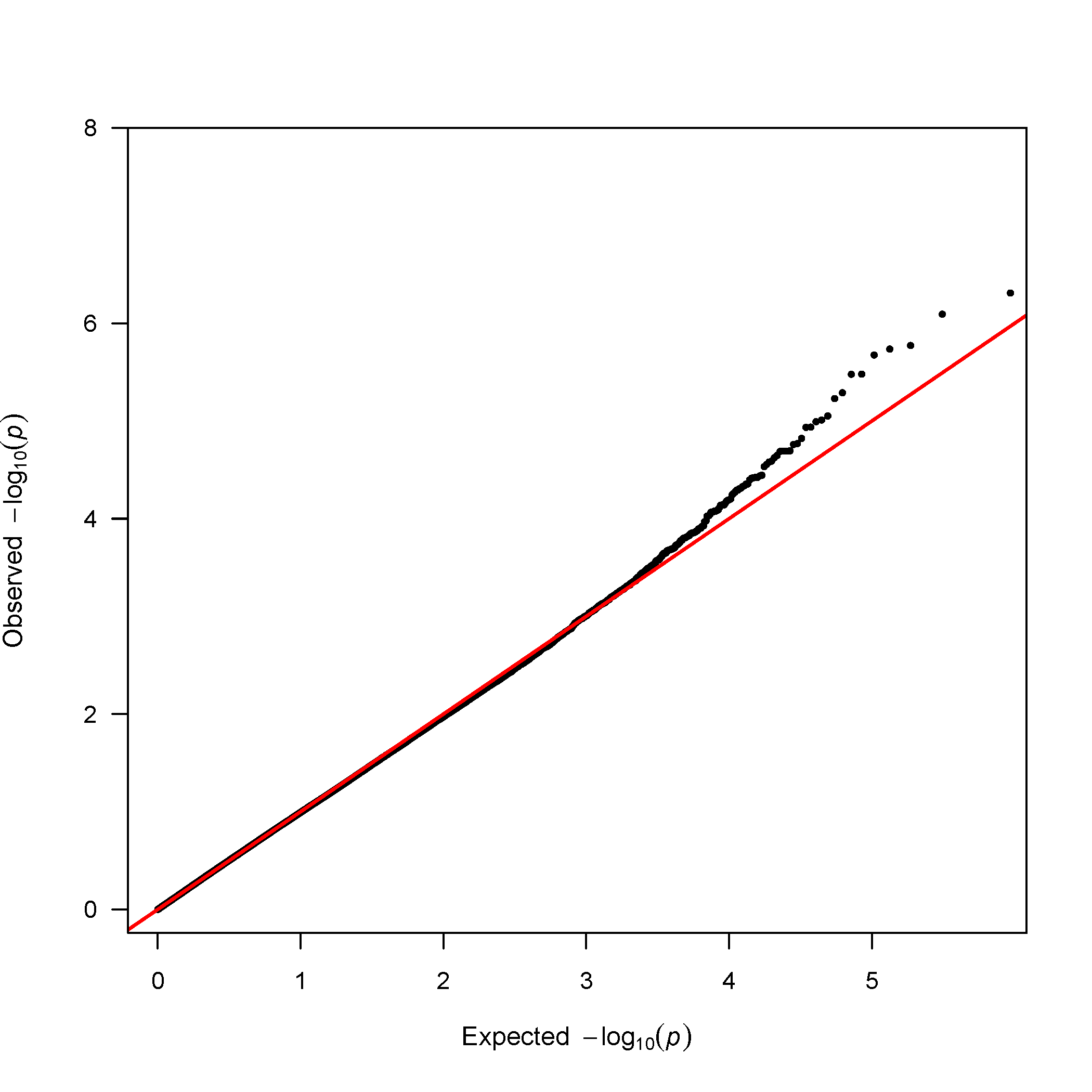

Supplement: S5 Fig — Plots the observed (Model1) versus expected -log10(p-values) under the null hypothesis of no association. (TIFF) [file pone.0156361.s005.tiff]

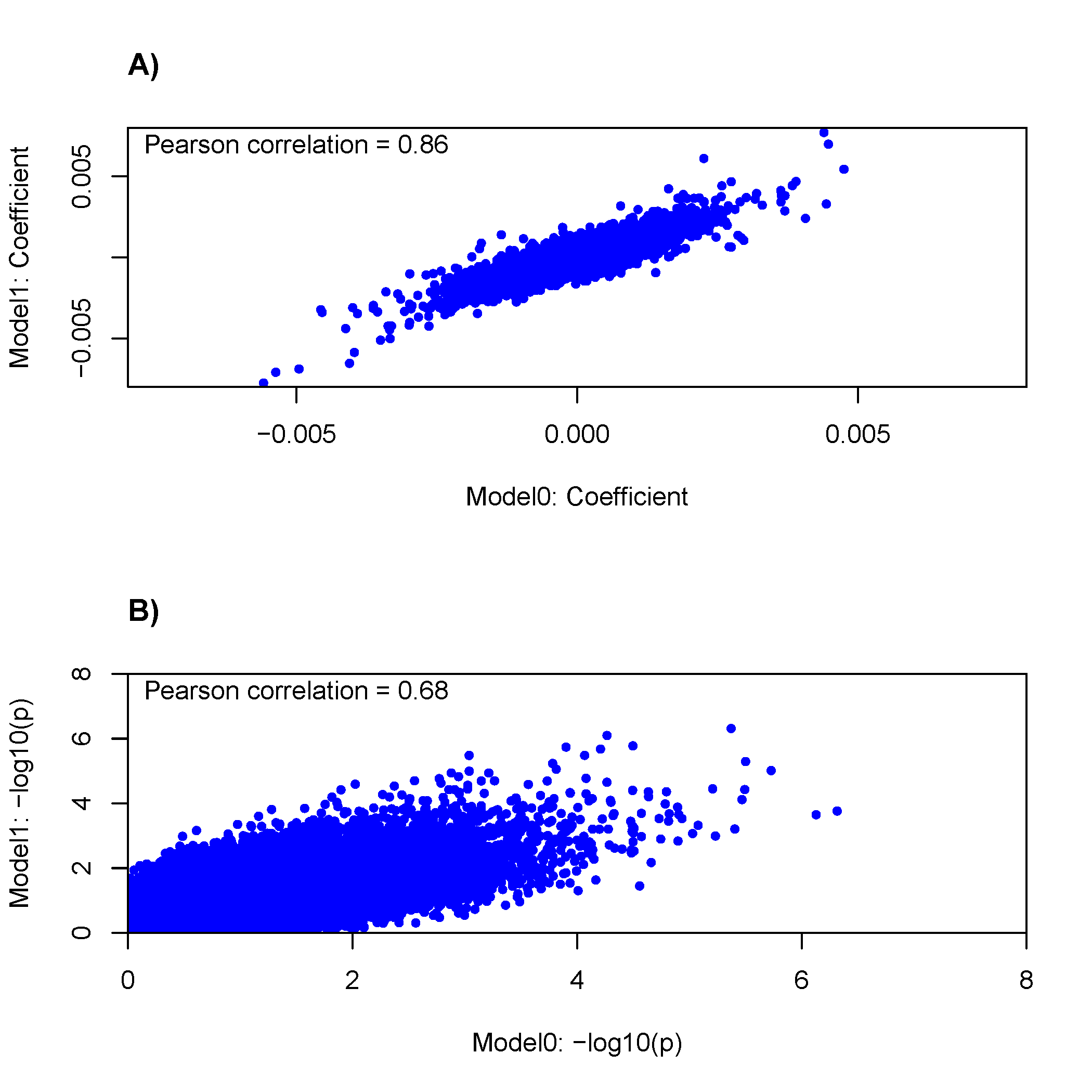

Supplement: S6 Fig — Model0: Methylation (β-value) = maternal age + batch + bisulfite conversion efficiency + infant’s birth year; Model1: Methylation (β-value) = maternal age + cleft + infant’s sex + batch + bisulfite conversion efficiency + infant’s birth year + infant’s birth weight + maternal alcohol use + maternal smoking + maternal education + parity, A) Comparison of the maternal age coefficient in Model0 versus Model1, B) Comparison of the maternal age –log10(P-value) in Model0 versus Model1. (TIFF) [file pone.0156361.s006.tiff]

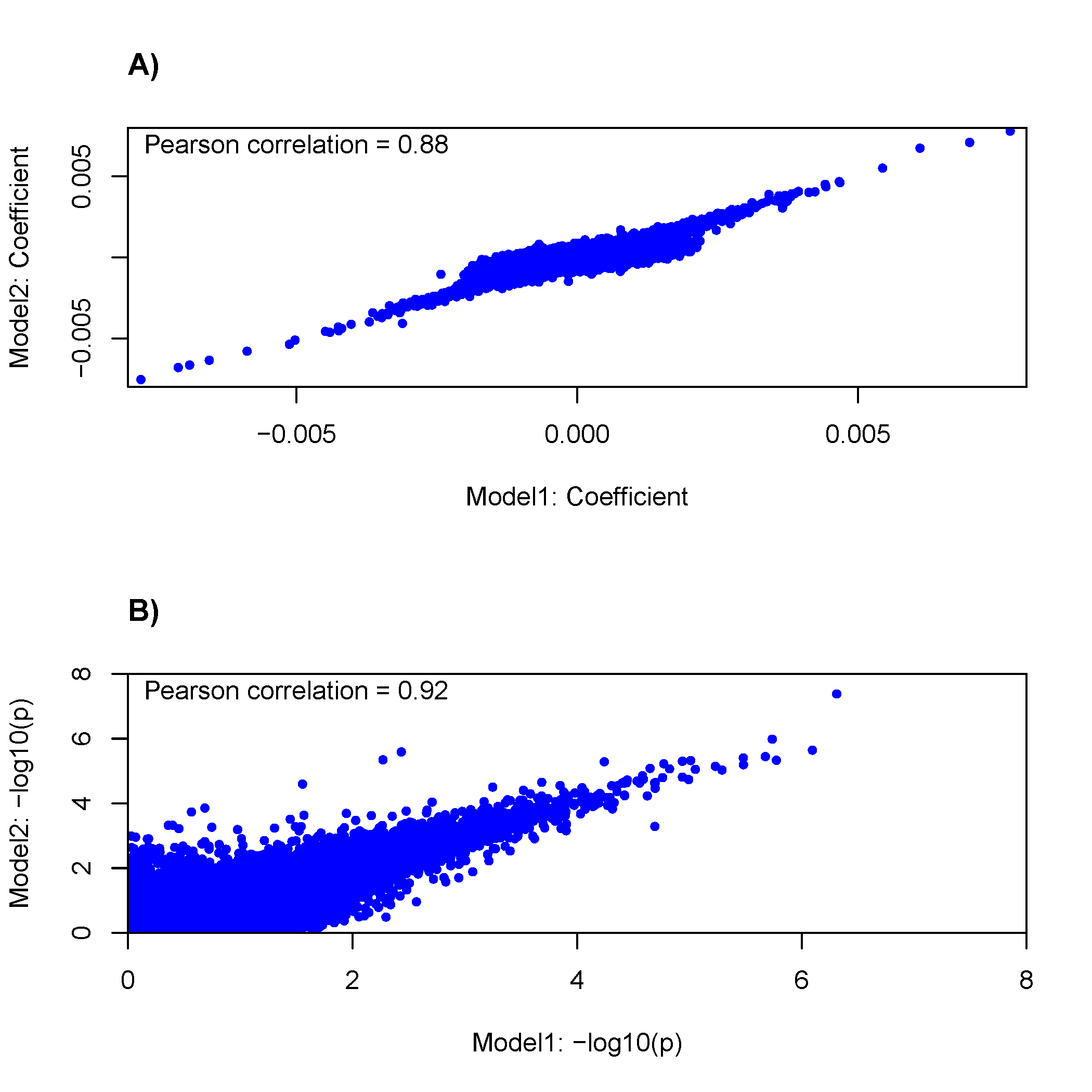

Supplement: S7 Fig — Model1: Methylation (β-value) = maternal age + cleft + infant’s sex + batch + bisulfite conversion efficiency + infant’s birth year + infant’s birth weight + maternal alcohol use + maternal smoking + maternal education + parity; Model2: Methylation (β-value) = maternal age + cleft + infant’s sex + batch + bisulfite conversion efficiency + infant’s birth year + infant’s birth weight + maternal alcohol use + maternal smoking + maternal education + parity + six leukocyte proportions (CD8+ T cells, CD4+ T cells, Natural killer cells, B cells, Monocytes, Granulocytes), A) Comparison of the maternal age coefficient in Model1 versus Model2, B) Comparison of the maternal age –log10(P-value) in Model1 versus Model2. (TIFF) [file pone.0156361.s007.tiff]
