# Supplementary material for: Maternal Age at Delivery Is Associated with an Epigenetic Signature in Both Newborns and Adults
Source: PLoS One. 2016 Jul 6;11(7):e0156361. doi: 10.1371/journal.pone.0156361 (PMC4934688; doi:10.1371/journal.pone.0156361)
Supplement: S1 Table — (DOCX) [file pone.0156361.s008.docx]

Table S1. Characteristics of mothers and infants in NFCS^a^

| **DESCRIPTION** | | | | **N^f^** | **%** |
| --- | --- | --- | --- | --- | --- |
| Infants | | | | 890 |  |
| Sex |  |  |  |  |  |
| Female | | | | 385 | 43.3% |
| Male | | | | 505 | 56.7% |
| Facial cleft status | | | |  |  |
| Control | | | | 473 | 53.1% |
| Case | | | |  |  |
| Cleft lip with or without cleft palate | | | | 274 | 30.8% |
| Cleft palate only | | | | 143 | 16.1% |
| Gestational age (days) | | | | 277.02 ± 12.86 | NA |
| Birth weight (grams) | | | | 3572.32 ± 610.71 | NA |
| Mothers | | | | 890 |  |
| Alcohol use^b^ | | | |  |  |
| 0 | | | | 568 | 64.5% |
| 1-3 | | | | 168 | 19.1% |
| 4-6 | | | | 57 | 6.5% |
| ≥ 7 | | | | 88 | 10.0% |
| Smoking^c^ | | | |  |  |
| Active | | | | 602 | 67.6% |
| Non-smokers | | | | 288 | 32.4% |
| Education | | | |  |  |
| Less than high school | | | | 118 | 13.3% |
| High school and above | | | | 772 | 86.7% |
| Parity | | | |  |  |
| 0 | | | | 368 | 41.3% |
| 1 | | | | 311 | 34.9% |
| 2 | | | | 156 | 17.5% |
| ≥ 3 | | | | 55 | 6.2% |
| Age at delivery (years) | | | | 29.64 ± 4.93 | NA |
| BMI (kg/m^2^)^d^ | | | | 24.66 ± 21.26 | NA |
| Folic acid supplement (µg)^e^ | | | |  |  |
| 0 | | | | 549 | 61.7% |
| 1-399 | | | | 192 | 21.6% |
| ≥ 400 | | | | 149 | 16.7% |
| Dietary folate (µg) | | | |  |  |
| 0-171 | | | | 301 | 33.8% |
| 172-214 | | | | 208 | 23.4% |
| 215-264 | | | | 188 | 21.1% |
| ≥ 265 | | | | 193 | 21.7% |
| Multivitamins^e^ | | | |  |  |
| No | | | | 588 | 66.1% |
| Yes | | | | 302 | 33.9% |

^a^Based on study population after sample exclusions (Ntotal=890)

^b^Total number of drinks during the first trimester

^c^Active smoking (≥ 1 cigarette/day) during 1st trimester

^d^Pre-pregnancy BMI

^e^During month before pregnancy and first two months of pregnancy

^f^When a continuous variable is presented, the mean ± standard deviation are listed

Abbreviations: BMI= body mass index, kg=kilogram, m=meter, µg=microgram, %=percentage, N=number
