# Supplementary material for: Maternal Age at Delivery Is Associated with an Epigenetic Signature in Both Newborns and Adults
Source: PLoS One. 2016 Jul 6;11(7):e0156361. doi: 10.1371/journal.pone.0156361 (PMC4934688; doi:10.1371/journal.pone.0156361)
Supplement: S2 Table — (DOCX) [file pone.0156361.s009.docx]

Table S2. Detailed *KLHL35* results across models^a^ in NFCS.

| **PROBE^b^** | **MEAN β-VALUE** | **Model0** | | | **Model1** | | | **Model2** | | |
| --- | --- | --- | --- | --- | --- | --- | --- | --- | --- | --- |
|  |  | **COEF** | **SE** | **P^c^** | **COEF** | **SE** | **P^c^** | **COEF** | **SE** | **P^c^** |
| cg05327192 | 0.93 | -0·00003 | 0.00008 | 0.73 | -0.0001 | 0.00010 | 0.50 | -0.0001 | 0.00010 | 0.52 |
| cg26561267 | 0.87 | 0.0001 | 0.00011 | 0.24 | 0.0002 | 0.00014 | 0.26 | 0.0001 | 0.00013 | 0.30 |
| cg02313829 | 0.99 | 0.000003 | 0.00002 | 0.86 | 0.000002 | 0.00002 | 0.91 | 0.000001 | 0.00002 | 0.96 |
| cg00388897 | 0.92 | -0.00004 | 0.00013 | 0.77 | -0.0001 | 0.00016 | 0.73 | -0.0001 | 0.00016 | 0.70 |
| cg02993825 | 0.96 | -0.00004 | 0.00004 | 0.29 | -0.00003 | 0.00004 | 0.46 | -0.00003 | 0.00004 | 0.54 |
| **cg06329735** | 0.37 | -0.005 | 0.00122 | **5.44E-05** | -0.007 | 0.00139 | **8.07E-07** | -0.007 | 0.00140 | **2.29E-06** |
| **cg05353869** | 0.37 | -0.005 | 0.00128 | **3.20E-05** | -0.007 | 0.00147 | **1.69E-06** | -0.007 | 0.00148 | **4.71E-06** |
| **cg04231094** | 0.54 | -0.002 | 0.00059 | **6.22E-05** | -0.003 | 0.00068 | **2.12E-06** | -0.003 | 0.00068 | **3.62E-06** |
| **cg10909185** | 0.30 | -0.006 | 0.00142 | **8.61E-05** | -0.008 | 0.00166 | **3.32E-06** | -0.008 | 0.00166 | **6.45E-06** |
| cg19149691 | 0.79 | -0.001 | 0.00039 | **2.56E-02** | -0.001 | 0.00042 | **6.22E-03** | -0.001 | 0.00041 | **6.14E-03** |
| cg11719952 | 0.89 | -0.001 | 0.00033 | **2.63E-02** | -0.001 | 0.00035 | **5.23E-03** | -0.001 | 0.00035 | **7.17E-03** |
| **cg16547529** | 0.52 | -0.001 | 0.00033 | **9.12E-04** | -0.001 | 0.00037 | **6.03E-04** | -0.001 | 0.00037 | **6.18E-04** |
| cg08160331 | 0.22 | -0.001 | 0.00027 | **7.03E-03** | -0.001 | 0.00032 | **5.78E-04** | -0.001 | 0.00032 | **1.24E-03** |
| cg26666804 | 0.05 | -0.0002 | 0.00012 | 0.08 | -0.0001 | 0.00014 | 0.56 | -0.00003 | 0.00014 | 0.81 |
| cg18113790 | 0.07 | -0.0001 | 0.00009 | 0.58 | -0.0001 | 0.00011 | 0.31 | -0.0001 | 0.00010 | 0.42 |
| cg23567562 | 0.02 | -0.00002 | 0.00005 | 0.71 | 0.000003 | 0.00006 | 0.96 | 0.00001 | 0.00006 | 0.91 |
| cg21555796 | 0.01 | 0.00001 | 0.00002 | 0.58 | 0.00002 | 0.00003 | 0.56 | 0.00002 | 0.00003 | 0.50 |
| cg12001148 | 0.04 | -0.00002 | 0.00005 | 0.76 | -0.000003 | 0.00007 | 0.96 | -0.00001 | 0.00007 | 0.92 |
| cg03934926 | 0.03 | 0.00005 | 0.00010 | 0.64 | 0.00002 | 0.00012 | 0.85 | 0.00003 | 0.00012 | 0.81 |
| cg16459103 | 0.19 | -0.0005 | 0.00023 | 0.05 | -0.0004 | 0.00025 | 0.08 | -0.0004 | 0.00025 | 0.15 |
| cg05456789 | 0.47 | -0.001 | 0.00069 | 0.06 | -0.001 | 0.00083 | 0.34 | -0.001 | 0.00083 | 0.37 |
| cg02343736 | 0.30 | -0.001 | 0.00033 | **4.03E-02** | -0.001 | 0.00040 | **8.89E-03** | -0.001 | 0.00038 | **5.76E-03** |
| cg20787146 | 0.61 | -0.001 | 0.00031 | 0.08 | -0.001 | 0.00033 | 0.09 | -0.001 | 0.00033 | 0.11 |

^a^Model0: adjusted only for technical factors (batch, bisulfite conversion efficiency, birth year); Model1: Model0 plus additional adjustment for cleft, infant's sex, infant’s birth weight, maternal alcohol use, maternal smoking, maternal education, and parity; Model2: Model1 plus additional adjustment for six leukocyte subtype proportions (CD8+ T cells, CD4+ T cells, Natural killer cells, B cells, Monocytes, Granulocytes)

^b^CpGs that are shown in bold were selected for replication analysis

^c^P-values < 0**·**05 are shown in bold

Abbreviations: BP=basepair, COEF=beta coefficient, SE=standard error of coefficient, P=p-value, NFCS=Norway Facial Clefts
