# Supplementary material for: Maternal Age at Delivery Is Associated with an Epigenetic Signature in Both Newborns and Adults
Source: PLoS One. 2016 Jul 6;11(7):e0156361. doi: 10.1371/journal.pone.0156361 (PMC4934688; doi:10.1371/journal.pone.0156361)
Supplement: S3 Table — (DOCX) [file pone.0156361.s010.docx]

Table S3. Detailed *KLHL35* Model1 results in NFCS - maternal age at delivery as quartiles.

| **PROBE^a^** | **CHR** | **BP^b^** | **2nd vs 1st quartile** | | | **3rd vs 1st quartile** | | | **4th vs 1st quartile** | | |
| --- | --- | --- | --- | --- | --- | --- | --- | --- | --- | --- | --- |
|  |  |  | **COEF** | **SE** | **P^c^** | **COEF** | **SE** | **P^c^** | **COEF** | **SE** | **P^c^** |
| cg05327192 | 11 | 75133593 | 0.001 | 0.00122 | 0.59 | -0.001 | 0.00128 | 0.66 | 0.000 | 0.00138 | 0.91 |
| cg26561267 | 11 | 75135383 | 0.001 | 0.00163 | 0.57 | 0.000 | 0.00171 | 0.80 | 0.001 | 0.00184 | 0.42 |
| cg02313829 | 11 | 75136574 | 0.000 | 0.00022 | 0.47 | 0.000 | 0.00023 | 0.20 | 0.000 | 0.00025 | 0.96 |
| cg00388897 | 11 | 75136655 | -0.001 | 0.00192 | 0.51 | 0.000 | 0.00200 | 0.90 | -0.002 | 0.00215 | 0.39 |
| cg02993825 | 11 | 75136839 | 0.000 | 0.00054 | 0.84 | 0.000 | 0.00056 | 0.42 | 0.000 | 0.00060 | 0.88 |
| **cg06329735** | 11 | 75139390 | -0.023 | 0.01688 | 0.18 | -0.035 | 0.01766 | 0.05 | -0.093 | 0.01901 | **1.17E-06** |
| **cg05353869** | 11 | 75139544 | -0.023 | 0.01787 | 0.19 | -0.039 | 0.01869 | **3.60E-02** | -0.098 | 0.02012 | **1.35E-06** |
| **cg04231094** | 11 | 75139680 | -0.009 | 0.00820 | 0.27 | -0.013 | 0.00858 | 0.13 | -0.045 | 0.00924 | **1.28E-06** |
| **cg10909185** | 11 | 75139736 | -0.038 | 0.01992 | 0.05 | -0.042 | 0.02084 | **4.25E-02** | -0.110 | 0.02243 | **1.05E-06** |
| cg19149691 | 11 | 75140541 | -0.001 | 0.00504 | 0.90 | -0.005 | 0.00527 | 0.32 | -0.016 | 0.00567 | **4.13E-03** |
| cg11719952 | 11 | 75140567 | -0.003 | 0.00418 | 0.44 | -0.009 | 0.00436 | **4.77E-02** | -0.014 | 0.00470 | **2.40E-03** |
| **cg16547529** | 11 | 75140681 | 0.001 | 0.00457 | 0.89 | -0.005 | 0.00478 | 0.25 | -0.015 | 0.00514 | **3.89E-03** |
| cg08160331 | 11 | 75140865 | -0.010 | 0.00386 | **1.17E-02** | -0.012 | 0.00403 | **2.84E-03** | -0.016 | 0.00436 | **2.55E-04** |
| cg26666804 | 11 | 75141627 | -0.001 | 0.00166 | 0.59 | 0.000 | 0.00174 | 0.90 | -0.001 | 0.00188 | 0.53 |
| cg18113790 | 11 | 75141722 | 0.000 | 0.00133 | 0.73 | -0.002 | 0.00138 | 0.12 | -0.001 | 0.00149 | 0.48 |
| cg23567562 | 11 | 75141735 | 0.000 | 0.00075 | 0.74 | 0.001 | 0.00078 | 0.20 | 0.000 | 0.00084 | 0.91 |
| cg21555796 | 11 | 75141793 | 0.001 | 0.00032 | 0.10 | 0.001 | 0.00034 | **3.88E-02** | 0.000 | 0.00037 | 0.27 |
| cg12001148 | 11 | 75141815 | 0.000 | 0.00079 | 0.80 | 0.001 | 0.00082 | 0.17 | 0.000 | 0.00089 | 0.91 |
| cg03934926 | 11 | 75141835 | 0.001 | 0.00144 | 0.36 | 0.001 | 0.00151 | 0.40 | 0.000 | 0.00162 | 0.89 |
| cg16459103 | 11 | 75141846 | -0.005 | 0.00304 | 0.08 | -0.008 | 0.00318 | **1.04E-02** | -0.007 | 0.00343 | **4.57E-02** |
| cg05456789 | 11 | 75141967 | -0.005 | 0.00985 | 0.59 | -0.008 | 0.01044 | 0.44 | -0.025 | 0.01119 | **2.60E-02** |
| cg02343736 | 11 | 75142012 | -0.003 | 0.00480 | 0.53 | -0.009 | 0.00502 | 0.06 | -0.017 | 0.00541 | **1.69E-03** |
| cg20787146 | 11 | 75142449 | 0.002 | 0.00397 | 0.54 | 0.003 | 0.00416 | 0.48 | -0.009 | 0.00448 | 0.05 |

Summary statistics: 1st quartile: Mean=23·5±2·2 (16·5-26·3); 2nd quartile: Mean=27·9±0·9 (26·3-29·6); 3rd quartile: Mean=31·0±1·0 (29·4-32·8); 4th quartile: Mean=36·2±2·5 (32·9-44·8)

^a^CpGs that are shown in bold were selected for replication analysis

^b^Physical location in basepairs (Human genome build GRCh37/hg19)

^c^P-values < 0·05 are shown in bold

Abbreviations: BP=basepair, COEF=beta coefficient, SE=standard error of coefficient, P=p-value, NFCS=Norway Facial Clefts
