# Supplementary material for: Maternal Age at Delivery Is Associated with an Epigenetic Signature in Both Newborns and Adults
Source: PLoS One. 2016 Jul 6;11(7):e0156361. doi: 10.1371/journal.pone.0156361 (PMC4934688; doi:10.1371/journal.pone.0156361)
Supplement: S4 Table — (DOCX) [file pone.0156361.s011.docx]

Table S4. Select *KLHL35* results for Model1 plus paternal age using the Norway Facial Clefts Study.

| **PROBE** | **Model1** | | | **Model1 + Paternal age** | | |
| --- | --- | --- | --- | --- | --- | --- |
|  | **COEF** | **SE** | **P^a^** | **COEF** | **SE** | **P^a^** |
| cg06329735 | -0.007 | 0.001 | **8.07E-07** | -0.009 | 0.002 | **5.88E-07** |
| cg05353869 | -0.007 | 0.001 | **1.69E-06** | -0.009 | 0.002 | **5.48E-06** |
| cg04231094 | -0.003 | 0.001 | **2.12E-06** | -0.004 | 0.001 | **9.19E-06** |
| cg10909185 | -0.008 | 0.002 | **3.32E-06** | -0.010 | 0.002 | **5.36E-06** |

^a^P-values < 0·05 are shown in bold.

Abbreviations: COEF=beta coefficient, SE=standard error of coefficient, P=p-value
