# Supplementary material for: Maternal Age at Delivery Is Associated with an Epigenetic Signature in Both Newborns and Adults
Source: PLoS One. 2016 Jul 6;11(7):e0156361. doi: 10.1371/journal.pone.0156361 (PMC4934688; doi:10.1371/journal.pone.0156361)
Supplement: S5 Table — (DOCX) [file pone.0156361.s012.docx]

Table S5. Replication of *KLHL35* maternal-age related DNA methylation changes in newborns and adults: a comparison of models^a^.

| **PROBE** | **STAT** | **NFCS** | | | **MoBa** | | | **SISTER STUDY** | | |
| --- | --- | --- | --- | --- | --- | --- | --- | --- | --- | --- |
|  |  | **Model0** | **Model1** | **Model2** | **Model0** | **Model1** | **Model2** | **Model0** | **Model1** | **Model2** |
| cg06329735 | COEF | -0.005 | -0.007 | -0.007 | -0.004 | -0.003 | -0.003 | -0.009 | -0.007 | -0.007 |
|  | P^b^ | **5.4E-05** | **8.1E-07** | **2.3E-06** | **4.3E-04** | **2.4E-02** | **2.4E-02** | **2.6E-03** | **2.0E-02** | **2.4E-02** |
| cg05353869 | COEF | -0.005 | -0.007 | -0.007 | -0.005 | -0.004 | -0.004 | -0.007 | -0.006 | -0.006 |
|  | P^b^ | **3.2E-05** | **1.7E-06** | **4.7E-06** | **1.1E-04** | **7.3E-03** | **7.4E-03** | **5.9E-03** | **3.4E-02** | **3.3E-02** |
| cg04231094 | COEF | -0.002 | -0.003 | -0.003 | -0.003 | -0.002 | -0.002 | -0.004 | -0.004 | -0.004 |
|  | P^b^ | **6.2E-05** | **2.1E-06** | **3.6E-06** | **1.8E-04** | **4.8E-03** | **5.4E-03** | **1.8E-03** | **1.5E-02** | **1.3E-02** |
| cg10909185 | COEF | -0.006 | -0.008 | -0.008 | -0.005 | -0.004 | -0.004 | -0.010 | -0.008 | -0.008 |
|  | P^b^ | **8.6E-05** | **3.3E-06** | **6.5E-06** | **1.0E-05** | **9.2E-04** | **8.1E-04** | **4.8E-03** | **3.8E-02** | **3.5E-02** |

^a^Model0: adjusted only for technical factors; Model1: Model0 plus additional adjustment for population selection factor, sex, and potential confounders; Model2: Model1 plus additional adjustment for leukocyte subtype proportions (for details on covariates, see Methods section)

^b^P-values < 0·05 are shown in bold. For the replication analysis in MoBa and the Sister Study, those sites that met a conservative Bonferroni correction for five tests are also underlined.

Abbreviations: COEF=beta coefficient, P=p-value, NFCS=Norway Facial Clefts, MoBa=Norwegian Mother and Child Cohort Study, NA=not applicable, STAT=statistic
