# Supplementary material for: Maternal Age at Delivery Is Associated with an Epigenetic Signature in Both Newborns and Adults
Source: PLoS One. 2016 Jul 6;11(7):e0156361. doi: 10.1371/journal.pone.0156361 (PMC4934688; doi:10.1371/journal.pone.0156361)
Supplement: S1 Text — (DOCX) [file pone.0156361.s013.docx]

**S1 Text. Supplemental Methods.**

**Discovery population: NFCS newborns**

As part of a standardized program of testing for phenylketonuria (PKU), heel stick blood samples were collected in glass capillary tubes from all newborns in Norway two to three days after delivery, frozen, and shipped to a central laboratory in Oslo for PKU testing and storage. Samples from cases and controls were subsequently retrieved and DNA was extracted for genetic analysis. Information on the mother’s and child’s date of birth was used to calculate maternal age at delivery and additional maternal and infant characteristics were obtained through mother’s questionnaire obtained three to four months after delivery. Study population characteristics (Appendix: Table S1) and the maternal age at delivery distribution are provided (Appendix: Fig. S1).

**Replication populations: MoBa newborns and Sister Study adults**

MoBa maternal age at delivery information was obtained from the Medical Birth Registry of Norway. Additional maternal and infant characteristics were obtained through the Medical Birth Registry of Norway or the maternal questionnaires in MoBa completed by the mother. The maternal age at delivery distribution in MoBa is shown in Fig. S1 (Appendix).

In the Sister Study, *in utero* exposure to diethystillbestrol (DES) was confirmed by both the participant and the participant’s mother [1]. Specifically, 100 women exposed to DES during fetal development and 100 unexposed women (frequency matched by age) were selected. Information on maternal age at delivery, as well as other maternal and participant characteristics, were based on either the participant’s response to a baseline questionnaire or, when available, the participant’s mother’s response to a separate questionnaire. The maternal age at delivery distribution in the Sister Study is shown in Fig. S1 (Appendix).

**Analysis: NFCS DNA methylation data**

In NFCS, DNA methylation probes were excluded as follows: 1) Presence on sex chromosomes, 2) 65 SNP probes that were part of the original array design, 3) Probes with a common SNP at the target site (Minor allele frequency > 0.05 based on 1000 Genomes European population [2]), 4) Greater than ten percent missing values across samples, and 5) Convergence issues with the statistical model. After exclusions, 465,525 probes remained for analysis. Eight samples were excluded for quality reasons as previously described[3], leaving 890 samples for analysis. The β-value was computed as the ratio between the methylated and total signal (Methylated signal / (Methylated signal + Unmethylated signal +100)) and used in the association analysis.

**Analysis: Epigenome-wide association analysis in NFCS**

Several models were considered: 1) Model0: Only adjusted for technical factors: batch (96-well plate), bisulfite conversion efficiency (see reference [3] for details), and infant’s birth year, 2) Model1 (Main model): Included all covariates in Model0 plus adjusted for the population selection factor (facial cleft status: control, cleft lip with or without cleft palate, and cleft palate only), infant’s sex, and additional covariates that could be potential confounders. Gestational age, infant’s birth weight, maternal alcohol use, maternal smoking, maternal education, parity, maternal body mass index (BMI), multivitamin use, dietary folate, and folic acid supplement use during pregnancy were all considered possible confounders, but only those associated with maternal age at delivery (p<0·05) were included in the model (infant’s birth weight, maternal alcohol use, maternal smoking, maternal education, and parity were included), and 3) Model2: Included all covariates in Model1 plus adjusted for six leukocyte subtype proportion estimates (CD8+ T cells, CD4+ T cells, Natural killer cells, B cells, Monocytes, Granulocytes) based on the method developed by Houseman and colleagues [4] as implemented in the R package, minfi (Reference dataset used: GSE35069 [5]). A comparison of models was conducted by examining the correlation between the p-values and beta coefficients across all CpGs tested (Appendix: Figs. S5-S6). Model1 was selected as the primary model due to differences between Model0 and Model1.

**Replication analyses: MoBa newborns and Sister Study adults**

As described above for the NFCS primary analysis, three models were assessed, although Model1 was considered the primary model. The models varied slightly by study population as follows: 1) Technical factors: For MoBa, adjustment for technical factors was performed during pre-processing steps rather than explicitly included within the model; For Sister Study, 96-well plate (batch) and bisulfite conversion intensities were adjusted for directly within the model, 2) Population selection factor: asthma status (MoBa) and breast cancer status (Sister Study), 3) Infant’s sex: Doesn’t apply to the Sister Study, 4) Maternal alcohol use: Not available in MoBa or the Sister Study, 5) Parity: Not available in the Sister Study, and 6) An additional covariate, age at blood draw, was included in the Sister Study for Models1 and 2.

**DNA methylation in single blastocysts (GSE51239)**

DNA methylation results for samples, GSM1240869 and GSM1240870, were extracted from 'GSE51239_hDev100bpMeFinal.txt' [6] for the *KLHL35* region of interest: chr11:75139390-75139736 (GRCh37/hg19). As described by Smith et al., the methylation level was estimated by the number of reads reporting a C, divided by the total number of reads (C or T). For the 100 base pair tiles, the methylation level was estimated by pooling reads for all CpGs that were covered by at least five reads [6].

**Supplemental References.**

1. Harlid S, Xu Z, Panduri V, D'Aloisio AA, DeRoo LA, et al. (2015) In utero exposure to diethylstilbestrol and blood DNA methylation in women ages 40-59 years from the sister study. PLoS One 10: e0118757.

2. Chen YA, Lemire M, Choufani S, Butcher DT, Grafodatskaya D, et al. (2013) Discovery of cross-reactive probes and polymorphic CpGs in the Illumina Infinium HumanMethylation450 microarray. Epigenetics 8: 203-209.

3. Markunas CA, Xu Z, Harlid S, Wade PA, Lie RT, et al. (2014) Identification of DNA Methylation Changes in Newborns Related to Maternal Smoking during Pregnancy. Environ Health Perspect 122: 1147-1153.

4. Houseman EA, Accomando WP, Koestler DC, Christensen BC, Marsit CJ, et al. (2012) DNA methylation arrays as surrogate measures of cell mixture distribution. BMC Bioinformatics 13: 86.

5. Reinius LE, Acevedo N, Joerink M, Pershagen G, Dahlen SE, et al. (2012) Differential DNA methylation in purified human blood cells: implications for cell lineage and studies on disease susceptibility. PLoS One 7: e41361.

6. Smith ZD, Chan MM, Humm KC, Karnik R, Mekhoubad S, et al. (2014) DNA methylation dynamics of the human preimplantation embryo. Nature 511: 611-615.
